# Sulforaphane reduces molecular response to hypoxia in ovarian tumor cells independently of their resistance to chemotherapy

**DOI:** 10.3892/ijo.2015.2987

**Published:** 2015-05-05

**Authors:** MICHAL PASTOREK, VERONIKA SIMKO, MARTINA TAKACOVA, MONIKA BARATHOVA, MARIA BARTOSOVA, LUBA HUNAKOVA, OLGA SEDLAKOVA, SONA HUDECOVA, OLGA KRIZANOVA, FRANCK DEQUIEDT, SILVIA PASTOREKOVA, JAN SEDLAK

**Affiliations:** 1 Cancer Research Institute, Slovak Academy of Sciences, Bratislava, Slovak Republic; 2 Institute of Virology, Slovak Academy of Sciences, Bratislava, Slovak Republic; 3 Centre of Molecular Medicine, Slovak Academy of Sciences, Bratislava, Slovak Republic; 4 Institute of Molecular Physiology and Genetics, Slovak Academy of Sciences, Bratislava, Slovak Republic; 5 Laboratory of Protein Signaling and Interactions, Interdisciplinary Cluster for Applied Genoproteomics, University of Liège, Sart-Tilman, Belgium; 6 Regional Centre for Applied Molecular Oncology, Masaryk Memorial Cancer Institute, Brno, Czech Republic

**Keywords:** sulforaphane, hypoxia, carbonic anhydrase IX, migration, chemoresistance

## Abstract

One of the recently emerging anticancer strategies is the use of natural dietary compounds, such as sulforaphane, a cancer-chemopreventive isothiocyanate found in broccoli. Based on the growing evidence, sulforaphane acts through molecular mechanisms that interfere with multiple oncogenic pathways in diverse tumor cell types. Herein, we investigated the anticancer effects of bioavailable concentrations of sulforaphane in ovarian carcinoma cell line A2780 and its two derivatives, adriamycin-resistant A2780/ADR and cisplatin-resistant A2780/CP cell lines. Since tumor microenvironment is characterized by reduced oxygenation that induces aggressive tumor phenotype (such as increased invasiveness and resistance to chemotherapy), we evaluated the effects of sulforaphane in ovarian cancer cells exposed to hypoxia (2% O_2_). Using the cell-based reporter assay, we identified several oncogenic pathways modulated by sulforaphane in hypoxia by activating anticancer responses (p53, ARE, IRF-1, Pax-6 and XRE) and suppressing responses supporting tumor progression (AP-1 and HIF-1). We further showed that sulforaphane decreases the level of HIF-1α protein without affecting its transcription and stability. It can also diminish transcription and protein level of the HIF-1 target, CA IX, which protects tumor cells from hypoxia-induced pH imbalance and facilitates their migration/invasion. Accordingly, sulforaphane treatment leads to diminished pH regulation and reduced migration of ovarian carcinoma cells. These effects occur in all three ovarian cell lines suggesting that sulforaphane can overcome the chemoresistance of cancer cells. This offers a path potentially exploitable in sensitizing resistant cancer cells to therapy, and opens a window for the combined treatments of sulforaphane either with conventional chemotherapy, natural compounds, or with other small molecules.

## Introduction

Tumors develop through the accumulation of genetic, epigenetic and somatic alterations that promote cell proliferation and survival. The expansive tumor growth then leads to architectural and physiological changes in the tumor tissue microenvironment, including a reduced oxygen delivery by the aberrant vasculature. Resulting hypoxia is one of the key drivers of cancer progression (1). It significantly affects crucial aspects of the tumor cell phenotype, such as metabolism, migration-invasion, dedifferentiation-stemness, and thereby supports metastasis (2). Moreover, hypoxia contributes to poor response of cancer patients to conventional anticancer therapies (3). In many tumor types, including ovarian carcinoma, hypoxia supports development of resistance to many currently used chemotherapeutic agents (4,5). Thus, it is important to search for new approaches and/or agents targeting hypoxic tumor cells in order to achieve better anticancer effects.

One of the recently emerging anticancer strategies is the use of natural dietary compounds, such as sulforaphane (SFN). SFN is a cancer-chemopreventive isothiocyanate found in cruciferous vegetables, namely in broccoli. Based on the growing experimental evidence, SFN acts through various molecular mechanisms that interfere with multiple oncogenic pathways and thereby induce anti-proliferative, anti-inflammatory, and pro-apoptotic responses in diverse tumor cell types (6–9). It also modulates metabolism of xenobiotics via induction of phase II detoxification enzymes (10). However, these studies were generally performed in normoxia and thus do not fully reflect the physiological situation in the tumor microenvironment. There are only few studies on SFN effect in hypoxic cancer cells (11–13). One of them, investigating the human prostate, tongue and colon carcinoma cells exposed to hypoxia showed that SFN can interfere with the HIF-1 pathway through decreasing the HIF-1α protein level and reducing the expression of the pro-angiogenic growth factor VEGF (12).

HIF-1 is a master transcription factor that orchestrates molecular responses to hypoxia. It is composed of two subunits, a constitutive β-subunit and an oxygen-sensitive α subunit. Oxygenation of cells leads to quick inactivation and proteasomal degradation of the HIF-1α subunit via a mechanism involving hydroxylation and pVHL-mediated ubiquitination. When oxygen level decreases these processes are inhibited, HIF-1α accumulates and dimerizes with HIF-1β to form the functional HIF-1 that binds a consensus HRE sequence in the promoters or enhancers of many genes mediating adaptive processes in hypoxic cells (14,15). The encoded proteins include VEGF as a mediator of tumor angiogenesis, GLUT-1 and glycolytic enzymes as mediators of metabolic reprogramming of cancer cells, CA IX as a mediator of acid-base balance in the tumors and many other regulatory molecules.

In this study, we focused on the effect of SFN in hypoxic ovarian carcinoma cells and in their chemoresistant variants. Ovarian cancer has the highest mortality among the gynecologic cancers. Most patients are diagnosed at a late stage and are usually treated by surgery followed by adjuvant chemotherapy. However, recurrence occurs in up to 75% of patients, who usually develop chemoresistance and eventually succumb to the disease. In ovarian carcinoma cells, hypoxia was correlated with poor prognosis, epithelial-mesenchymal transition, invasiveness, metastasis and stem-like phenotype (4,16,17). Moreover, HIF-1 target genes were proposed to predict increased resistance to chemotherapy and poor overall survival of ovarian cancer patients (18).

We found that in A2780 ovarian carcinoma cells exposed to low oxygen, SFN modifies the transcriptional program driven by several pathways related to hypoxia and oncogenic signaling. We specifically focused on the HIF pathway and demonstrated that SFN can reduce the protein levels of HIF-1α in hypoxic A2780 cells without affecting its transcription and stability. This results in diminished promoter activation, transcription and protein levels of the HIF-1 transcriptional target CA IX. Moreover, these effects can be recapitulated in ovarian cancer cell variants A2780/ADR and A2780/CP resistant to adriamycin and cisplatin, respectively, suggesting that chemoresistance cannot abolish the SFN-mediated down-modulation of adaptive responses to hypoxia. Finally, we showed that these effects of SFN lead to reduced invasiveness of all three ovarian cell lines supporting the view that SFN consumption may have beneficial anticancer effects also in the advanced cancer stages characterized by the presence of hypoxic tumor regions and associated aggressive tumor features.

## Materials and methods

### Cell lines, reagents and culture conditions

The human ovarian cancer cell line A2780 and derived cell lines resistant to adriamycin A2780/ADR and cisplatin A2780/CP were described earlier (19,20). The cells were cultured in Dulbecco’s modified Eagle’s medium supplemented with 10% fetal calf serum (Bio-Whittaker, Verviers, Belgium) and 40 mg/ml gentamicin (Lek, Ljubljana, Slovenia) in a humidified atmosphere with 5% CO_2_ at 37°C. Exposure to hypoxia was performed in an anaerobic workstation (Ruskin Technologies, Bridgend, UK) in 2% O_2_, 5% CO_2_, 10% H_2_, and 83% N_2_ at 37°C. Alternatively, hypoxia was mimicked by 1 mM dimethyloxalylglycine. Sulforaphane was obtained from Sigma-Aldrich (St. Louis, MO, USA) and used at 2.5–10 μM depending on the experimental setting. The cells were first pre-treated with SFN for 4 h and then continuously incubated with SFN in normoxia and/or hypoxia for additional 24 h or for longer periods.

### PCR analysis

Total RNA was extracted using the Instapure reagent (Eurogentec, Seraing, Belgium) as recommended by the manufacturer. Three micrograms of RNA was transcribed with a High-Capacity cDNA Reverse Transcription kit (Applied Biosystems, Foster City, CA) using random heptameric primers. Quantitative real-time PCR was performed on a StepOne Real-Time PCR System (Applied Biosystems) using Power SYBR Green PCR Master Mix (Applied Biosystems) and gene-specific primers (*CA9, HIF-1α*) and primers for *β-actin* that served as an internal standard. The primers were as follows: CA9 sense: 5′-CCGAGCGACGCAGCCTTTGA-3′ and CA9 antisense: 5′-GGCTCCAGTCTCGGCTACCT-3′; HIF-1α sense 5′-GCT TGGTGCTGATTTGTGAACC-3′, HIF-1α antisense 5′-GCA TCCTGTACTGTCCTGTGGTG-3′; β-actin sense: 5′-TCCTC CCTGGAGAAGAGCTA-3′ and β-actin antisense: 5′-ACAT CTGCTGGAAGGTGGAC-3′. PCR was performed using DreamTaq™ Green PCR Master Mix (Fermentas, St. Leon-Rot, Germany) and the same primers as shown above.

### Promoter analysis

Human *CA9* promoter construct pGL3-CA9 was generated by an insertion of PCR-amplified −174/+37 *CA9* genomic fragment upstream of the firefly luciferase gene in pGL3-Basic luciferase reporter vector (Promega, Madison, WI, USA). pRL-TK Renilla vector (Promega) served as a transfection efficiency control. A2780 cells were plated into 35-mm Petri dishes to reach approximately 70% monolayer density on the next day. Transient transfection was performed with 1 μg of pGL3-CA9 plasmid and 100 ng of pRL-TK plasmid using Turbofect reagent (ThermoFisher Scientific) according to the manufacturer’s recommendations. One day later, transfected cells were trypsinized and plated in triplicates into 24-well plates. Transfected cells were allowed to attach overnight, and then transferred to hypoxia for additional 24 h. SFN was added 4 h before the transfer to hypoxia. Reporter gene expression was assessed using the Dual Luciferase Reporter Assay System (Promega), and the luciferase activity was normalized against the Renilla activity.

### Western blot analysis

The cells were washed with PBS and disrupted in lysis buffer containing 1% Triton X-100, 150 mM NaCl, 50 mM Tris (pH 7.5), 0.5% Nonidet P-40, 50 mM NaF, and complete protease inhibitor cocktail (Roche, Mannheim, Germany). Protein concentrations were determined by bicinchoninic acid assay (Pierce Biotechnology, Rockford, IL, USA) according to the manufacturer’s instructions. Total protein extracts (50–100 mg/lane) were separated by SDS-PAGE under reducing conditions and blotted onto polyvinylidene fluoride membranes (Immobilon; Millipore, Billerica, MA, USA). Membranes were treated for 1 h in blocking buffer and then incubated either for 1 h with specific antibodies against CA IX (in-house generated M75 in blocking buffer, dilution 1:2), HIF-1α (dilution 1:250; BD Transduction Laboratories, San Jose, CA, USA), GLUT-1 (dilution 1:1000; Cell Signaling Technology, Danvers, MA, USA), and actin (dilution 1:1000; Santa Cruz Biotechnology, Santa Cruz, CA, USA). All membranes were then washed four times for 10 min with the washing buffer (PBS containing 0.2% Nonidet P-40 or 0.1% Tween-20), followed by the incubation with an appropriate secondary antibody conjugated with horseradish peroxidase (Dako, Glostrup, Denmark) for 1 h. After additional washing step, all immunoblots were developed with the ECL detection system.

### Flow cytometry

Cells were harvested in concentration of 1×10^6^ cells/sample and washed in PBS. Labeling was performed in 50 μl of cell suspension with 50 μl of mouse monoclonal antibody M75 for 30 min. Mouse monoclonal antibody against CD45 was used as a negative isotype control. Cells were then washed and labeled with goat anti-mouse F(ab’)2 antibody conjugated with FITC for 30 min at room temperature and analyzed on flow cytometer Coulter Epics Altra. Data were analyzed with FCS Express version 3.0 (De Novo Software, Ontario, Canada).

For assessment of cell viability, the cells were incubated with propidium iodide at a final concentration of 5 μg/ml for 5 min at room temperature. The samples were analyzed using a Guava EasyCyte Plus flow cytometer with Guava Express Pro 2.2.3 software (Millipore).

### Cignal assay

Cignal^®^™ Cell-based Multi-Pathway Activity Assay (SABioscience, Frederick, MD, USA) was performed according to instructions of the manufacturer. Dual-luciferase results were calculated for each transfectant and analyzed by the data analysis software (SABioscience). Changes in the activity of each signaling pathway were determined by comparing the normalized luciferase activities of the reporter in treated vs. untreated transfected cells.

### Cell proliferation and migration assays using real-time cell analyzer system (RTCA)

For the proliferation assay, 4×10^4^ A2780 ovarian cancer cells and their chemoresistant variants were seeded into each well of an E-plate 16 (Roche) in DMEM containing 5 μM sulforaphane or DMSO (control samples). For the migration assay, the cells were first pre-treated with sulforaphane or DMSO for 24 h in hypoxia and then seeded into the upper chamber of the CIM-plate 16 (Roche) in the medium containing 0.1% FCS and 5 μM sulforaphane. A chemotactic signal for the cell movement was provided by supplying 10% FCS into the lower chamber. The plates were placed into the real-time cell analyzer (RTCA, xCELLigence, Roche), performing an impedance-based, label-free monitoring of cell proliferation and migration. RTCA analyzer was placed in a hypoxic cabinet with the O_2_ controller (2% O_2_, COY Laboratory Product, Grass Lake, MI, USA). Data were collected every 15 min during the entire period of measurement and were presented as a dimensionless parameter called the cell index (CI; calculated as a relative change in the measured electrical impedance), graphs show the average of triplicate wells.

### Measurements of intracellular and extracellular pH

Intracellular pH (pHi) was measured using the fluorescent probe 2′,7′-biscarboxyethyl-5,6-carboxyfluorescein (BCECF; Sigma-Aldrich). SFN-treated cells, untreated controls and cells for calibration curve were plated on 6-well plates and loaded with 8.2 μM BCECF and 5% pluronic acid in PBS buffer, pH 7.48 for 30 min at 37°C in the dark. Afterwards, the cells were washed with PBS buffer and measured. Calibration was performed on untreated cells using PBS/HEPES buffers with different pH values (7.61, 7.48, 7.03, 6.52, 6.32 and 6.01). The fluorescence was excited at 489 nm and measured at 525 nm on the fluorescence scanner BioTek (BioTek, Germany). The pHi signal was calibrated by adding 10 μM of nigericin (Sigma-Aldrich) with 130 mM of KCl. pHi values for samples were calculated from the calibration curve. Extracellular pH was measured in cell culture media as described earlier (21).

### Statistical analysis

Results were analyzed by two-tailed unpaired t-test (Student’s t-test), and P<0.05 was considered significant.

## Results

### SFN affects hypoxia-induced and oncogenic molecular pathways in A2780 ovarian carcinoma cells

**Figure 1:**
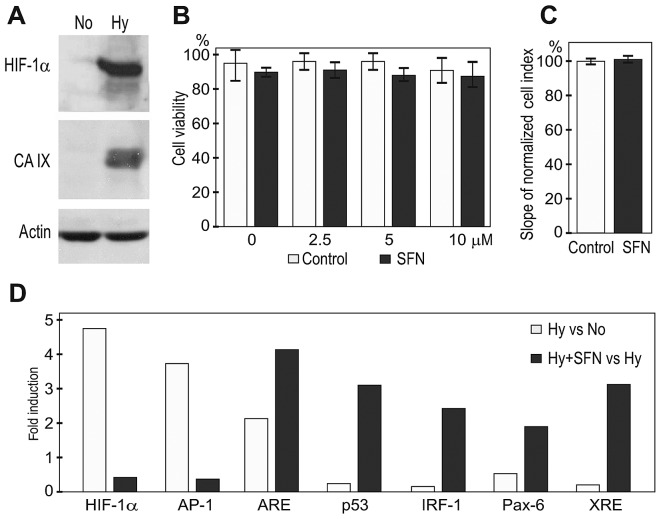
Molecular and cellular response of A2780 cells to hypoxia and SFN. (A) Western blot analysis of HIF-1α and CA IX expression in A2780 cells. The blot shows that these proteins are absent in normoxia (No) but induced in response to hypoxia (Hy). Actin serves as a loading control. (B) Flow cytometric analysis of the viability of hypoxic vs. normoxic A2780 cells (control) and the parallel normoxic vs. hypoxic samples treated with increasing concentrations of SFN as described in Materials and methods. The graph depicting percentage of live (propidium iodide-negative) cells in population indicates only insignificant decrease in cell viability in all treated samples. (C) Real-time analysis of proliferation of the hypoxic A2780 cells treated with 5 μM SFN for 48 h compared with the non-treated controls. The graph shows the slope of cell index normalized to 6 h time point after plating at which time all cells were attached to the bottom of the wells of the impedance plate and hypoxia was settled at 2%. Data indicate that SFN treatment did not affect the proliferation of hypoxic A2780 cells. (D) Cell-based dual luciferase reporter assay of hypoxic vs. normoxic A2780 cells and hypoxic A2780 cells vs. cells treated with 5 μM SFN revealed hypoxia- and SFN-induced alterations of several signal transduction pathways leading to changes in transactivation activities of the transcription factors indicated in the graph. In principle, SFN downregulated pro-oncogenic pathways (represented by HIF-1α and AP-1) and upregulated anti-oncogenic pathways (such as ARE/NRF2, p53, IRF-1, Pax-6 and XRE).

First we investigated how A2780 ovarian carcinoma cells respond to hypoxia and SFN in a broader context of molecular pathways. We exposed A2780 cells for 24 h to 2% of atmospheric oxygen, which corresponds to moderate hypoxia typical for tumor cells present in broader perinecrotic areas less distant from the blood vessels. Western blot analysis of extracts from normoxic vs. hypoxic A2780 cells revealed a hypoxia-related accumulation of HIF-1α protein and its target CA IX suggesting that these cells are sensitive to reduced oxygen and react by induction of a canonical HIF response (Fig. 1A). The A2780 cells grown in a confluent monolayer were then treated with SFN for 28 h, including 4 h pre-treatment in normoxia followed by a 24 h treatment in hypoxia. Cells treated with 2.5, 5 and 10 μM concentrations of SFN were subjected to flow cytometric analysis to assess their viability. As shown on Fig. 1B, hypoxia alone induced 10% cell death of A2780 cells, which was only slightly increased by SFN treatments. Moreover, a real-time monitoring of the proliferation of A2780 cells did not show any pronounced inhibitory effect of 5 μM SFN either in normoxia (not shown), or in hypoxia (Fig. 1C).

Since the concentration of about 5 μM SFN is achievable *in vivo* (22), we decided to use this SFN concentration in the subsequent experiments. A cell-based reporter assay confirmed the hypoxia-related activation of the HIF pathway (Fig. 1D). In addition, exposure of A2780 cells to hypoxia led to the activation of the MAPK/JNK pathway executed by the AP-1 transcription factor, which was previously linked to hypoxia and VHL deficiency (23,24). SFN treatment considerably reduced the reporter transactivation via HIF and AP-1 pro-oncogenic pathways. On the other hand, five other pathways involved in the negative control of tumor growth were considerably upregulated by SFN during hypoxia, including the pathways resulting in activation of ARE/NRF2, p53, IRF-1, Pax-6 and XRE-driven transcription (Fig. 1D).

### SFN reduces the HIF-1α level and activity in hypoxic A2780 cells without affecting its stability

**Figure 2:**
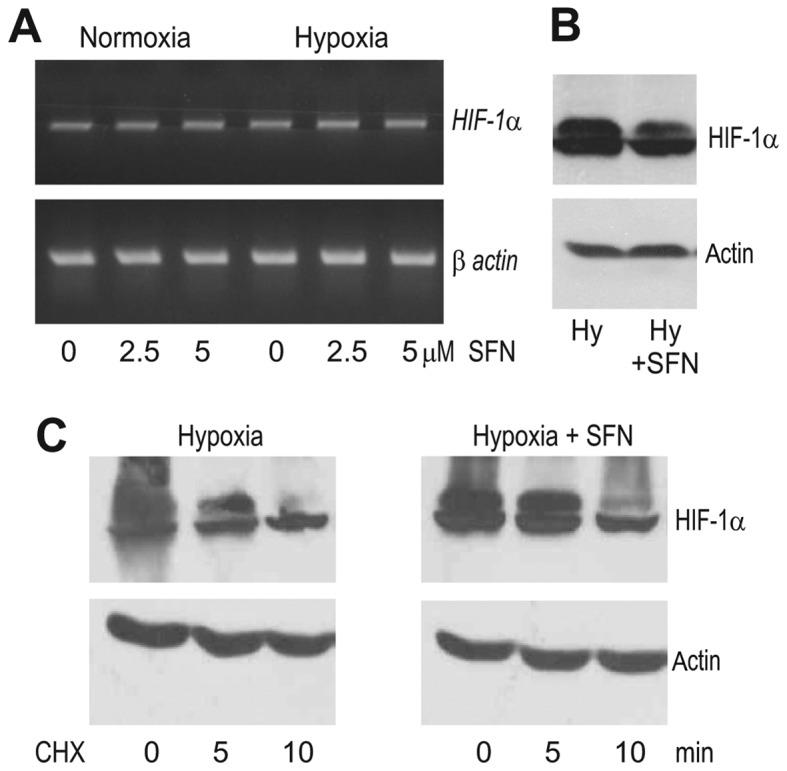
Effect of SFN on HIF-1α transcription, protein level and stability in A2780 cells. (A) Reverse-transcription PCR analysis of the HIF-1α transcription in normoxic and hypoxic A2780 cells in the absence and the presence of SFN. β-actin was used as standard. SFN treatment did not change the levels of the *HIF-1α* transcript. (B) Western blot analysis of HIF-1α protein levels in non-treated hypoxic A2780 cells (Hy) and in hypoxic A2780 cells treated with 5 μM SFN (Hy+SFN). (C) Western blot analysis of HIF-1α protein stability in non-treated (Hypoxia) and in 5 μM SFN-treated hypoxic A2780 cells (Hypoxia+SFN) in the presence of 20 μg/ml cycloheximide (CHX). SFN treatment did not affect the HIF-1α level suggesting that it did not decrease the HIF-1α protein stability.

We then further followed the HIF-1α response to SFN under hypoxia. First, we analyzed the SFN effect on the HIF-1α mRNA level and found no significant difference between SFN-treated and control A2780 cells exposed to hypoxia, suggesting that SFN did not affect the transcription of the *HIF-1α* gene (Fig. 2A). In contrast, the western blot analysis showed a considerably reduced level of the HIF-1α protein in the hypoxic A2780 cells subjected to SFN treatment when compared to the control hypoxic cells (Fig. 2B). Since the abundance of the HIF-1α protein can be regulated on the level of translation and/or degradation, we analyzed the stability of the HIF-1α protein in hypoxic conditions following the inhibition of translation by 20 μg/ml cycloheximide (CHX). However, the levels of the HIF-1α protein were similar in the hypoxic A2780 cells incubated with CHX for 10 min whether or not they were pre-treated with SFN (Fig. 2C), indicating that SFN did not induce HIF-1α degradation. This supports the view that SFN acts through the suppression of HIF-1α translation.

**Figure 3:**
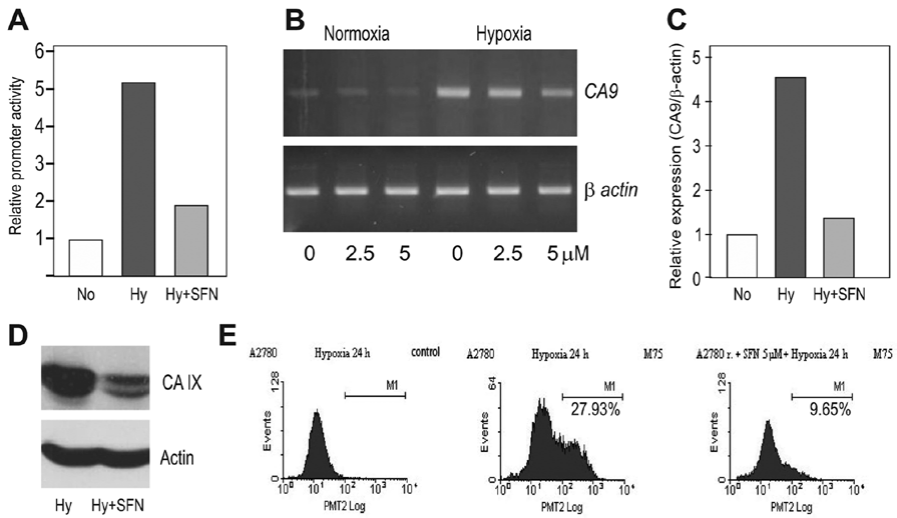
Effect of SFN on expression of the HIF-1α target CA IX. (A) Analysis of the *CA9* promoter activity in normoxic and hypoxic A2780 cells and in hypoxic A2780 cells treated with SFN. A2780 cells were co-transfected with pGL3-CA9 and pRL-TK plasmids, incubated for 24 h in normoxia (No), hypoxia (Hy) and in hypoxia with 5 μM SFN (Hy+SFN), and analyzed by dual luciferase assay. The data show that in hypoxic A2780 cells, SFN reduced the *CA9* promoter activity almost to the normoxic level. (B) RT-PCR analysis of the *CA9* transcription in normoxic and hypoxic A2780 cells in the absence and the presence of SFN. β-actin was used as standard. *CA9* transcription was induced by hypoxia and SFN treatment led to its dose-dependent decrease. (C) This reducing effect of SFN was confirmed by quantitative PCR analysis. (D) Western blot analysis of CA IX protein levels in non-treated hypoxic A2780 cells (Hy) and in hypoxic A2780 cells treated with 5 μM SFN (Hy+SFN). (E) Flow cytometric analysis of the CA IX expression in the population of hypoxic A2780 cells. The histograms show hypoxic cells incubated without the primary antibody (left), hypoxic cells labeled with the CA IX-specific monoclonal antibody M75 (middle), and SFN-treated hypoxic cells labeled with M75 (right). SFN treatment resulted in the reduced proportion of the CA IX-positive cells.

In the next step, we analyzed the effects of SFN on the HIF-1α downstream target CA IX. In agreement with the reduced HIF-1α protein level, SFN was able to diminish the hypoxia-induced activation of the *CA9* gene promoter almost to its normoxic value as determined by the dual luciferase assay (Fig. 3A). It could also reduce the level of the corresponding transcript as evident from the PCR analysis (Fig. 3B and C) and finally, decrease the level of the CA IX protein as seen in the western blot analysis (Fig. 3D). Moreover, the flow cytometric analysis revealed that SFN treatment led to a reduced proportion of the CA IX-positive tumor cell subpopulation of the hypoxic A2780 cells (Fig. 3E).

### SFN decreases the expression of HIF-1α and its targets CA IX and GLUT-1 in chemoresistant A2780/CP and A2780/ADR cells

**Figure 4:**
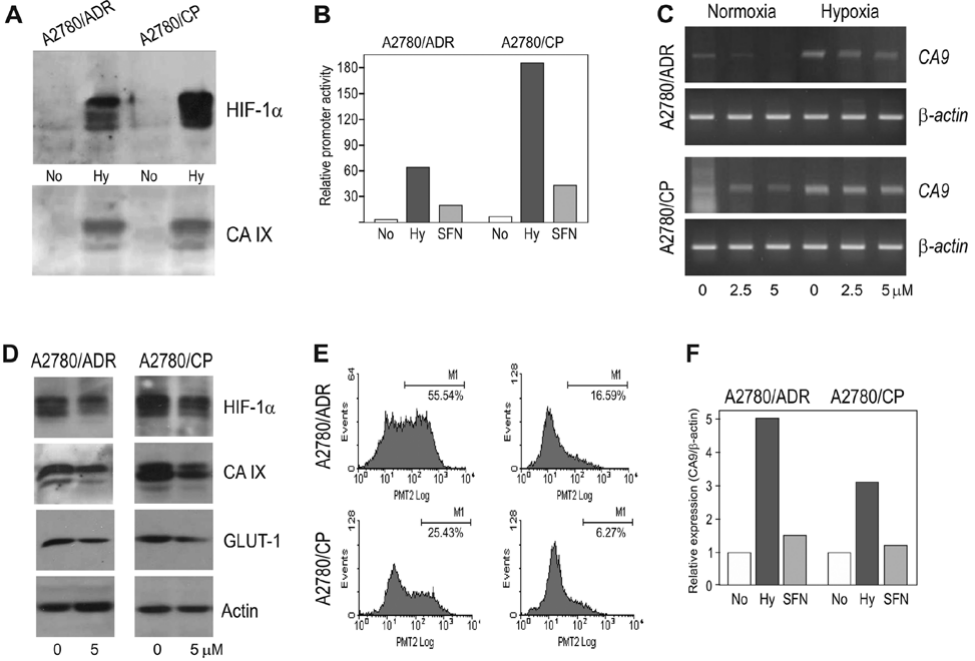
Responses of chemoresistant A2780/ADR and A2780/CP cells to hypoxia and SFN. (A) Western blot analysis of HIF-1α and CA IX expression in adriamycin-resistant A2780/ADR cells and in cisplatin-resistant A2780/CP cells. The blots show that these proteins are absent in normoxia (No) but induced in response to hypoxia (Hy). (B) Dual luciferase analysis of the *CA9* promoter activity in A2780/ADR and A2780/CP in normoxia, hypoxia and in hypoxia with SFN. The data show strong increase in the *CA9* promoter activity in both cell variants in hypoxia and reduction in the promoter activity in hypoxic cells treated with SFN. (C) RT-PCR analysis of the *CA9* transcription in normoxic and hypoxic A2780/ADR and A2780/CP cells in the absence and the presence of SFN. β-actin was used as standard. *CA9* transcription was induced by hypoxia, and SFN treatment led to its dose-dependent decrease. (D) Western blot analysis of non-treated hypoxic A2780/ADR and A2780/CP cells (Hy) and of hypoxic cells treated with 5 μM SFN (Hy+SFN) for expression of HIF-1α and its targets CA IX and GLUT-1. Actin was used as a loading control. SFN treatment led to decreased levels of all three analyzed proteins. (E) Flow cytometric analysis of the CA IX expression in the population of hypoxic A2780/ADR and A2780/CP cells labeled with the M75 antibody. The histograms show hypoxic cells (left) and SFN-treated hypoxic cells (right). SFN treatment resulted in a reduced proportion of the CA IX-positive cells. (F) The graph illustrates the cytometric data as the relative CA IX expression values.

Drug resistance is one of the key obstacles in therapy of ovarian cancer, and hypoxia is known to support this phenomenon by activation of molecular mechanisms of multiple drug resistance as well as by selection of cells that are less responsive to drug treatment (5). Therefore, we evaluated the effect of SFN in the chemoresistant variants of A2780 cells under hypoxic conditions using the A2780/CP cell line resistant to cisplatin (expressing the MRP1 gene) and A2780/ADR cell line resistant to adriamycin (expressing the MDR1 gene). Of note, these chemoresistant cell lines showed increased HIF-1α protein levels when exposed to 2% hypoxia similarly to parental chemosensitive A2780 cells (Fig. 4A). This was associated also with the increased *CA9* promoter activation (Fig. 4B), increased induction of the *CA9* transcript (Fig. 4C) and with the elevated CA IX protein expression (Fig. 4A).

Noteworthy, SFN was able to reduce the molecular response to hypoxia in both cell lines in an extent similar to that in parental A2780 cells, although the inhibitory effect did not go back to the normoxic values either at the level of *CA9* mRNA or at the protein levels of both HIF-1α and its CA IX and GLUT-1 downstream targets (see Fig. 4B–D). Moreover, in both chemoresistant cell lines SFN reduced the proportion of the CA IX-positive subpopulation of cells similarly to that observed in the parental A2780 cell line (Fig. 4E and F). Altogether, these data suggest that SFN can exhibit its anticancer effect also in the chemoresistant cell lines.

### SFN affects pH regulation and reduces migration of hypoxic A2780 cells as well as their chemoresistant variants

**Figure 5:**
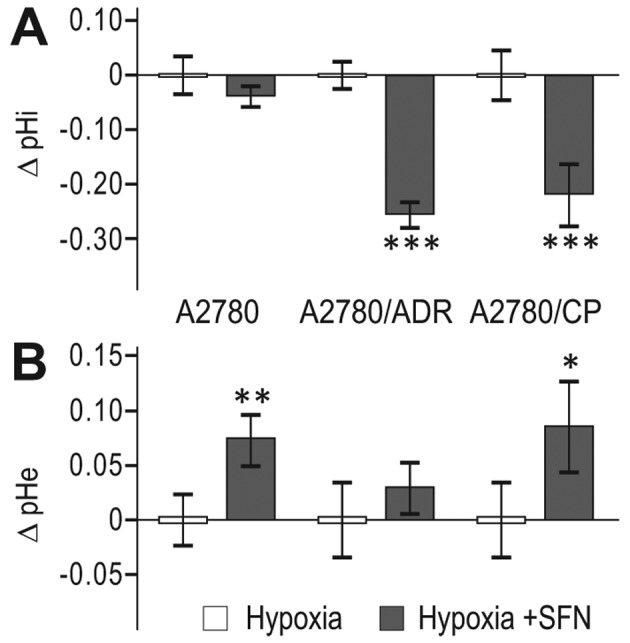
Effect of SFN on pH regulation in A2780 cells and their chemo-resistant variants. (A) SFN-induced changes in intracellular pH (pHi). The cells were loaded with SNARF and analyzed as described in Materials and methods. The graph shows differences in pHi in all three analyzed cell lines that were plated in triplicates and treated with SFN compared to control non-treated cells. (B) SFN-induced changes in extracellular pH (pHe) measured in culture media of cells exposed to hypoxia for 24 h (Hy) and of hypoxic cells treated with SFN (Hy+SFN). The graph shows differences in pHe between SFN treated and non-treated cells. ^*^p<0.05, ^**^p<0.01, and ^***^p<0.001.

Since CA IX, cooperating with ion transporters and exchangers, is functionally implicated in acidification of extracellular pH (pHe) and in maintenance of neutral/slightly alkaline intracellular pH (pHi), we evaluated the pH changes in response to 24 h SFN treatment. We found decreased pHi (Fig. 5A) and increased pHe (Fig. 5B) in SFN-treated hypoxic cells compared to non-treated hypoxic controls, suggesting that SFN interferes with the capacity of the pH regulating machinery of the parental A2780 cells as well as of both A2780/ADR and A2780/CP chemoresistant cell lines to maintain the proper acid-base balance in hypoxia.

**Figure 6:**
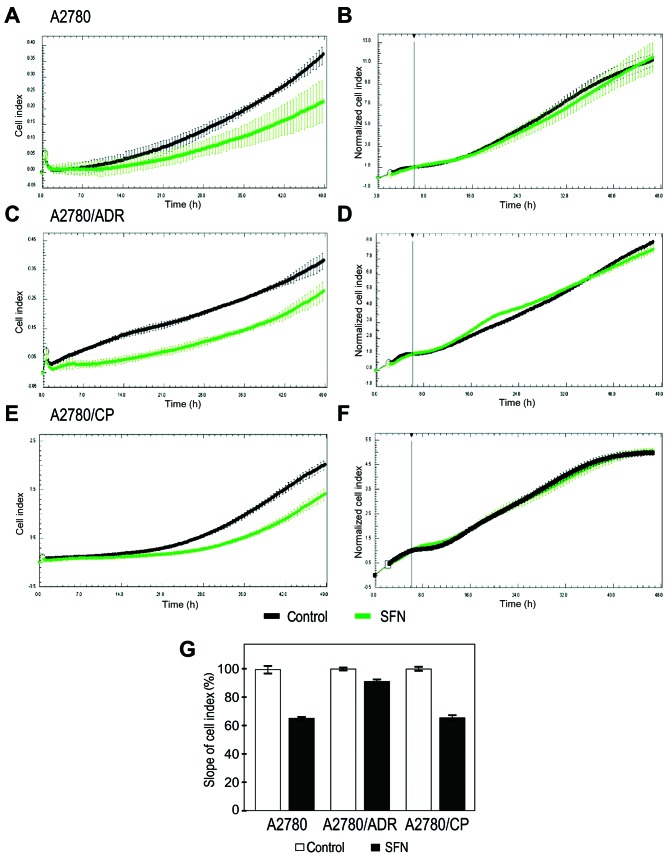
Effect of SFN on migration of A2780 cells and their chemoresistant variants. Real-time impedance-based measurement of migration ability (A, C and E) and proliferation (B, D and F) of SFN-treated vs. non-treated hypoxic cells A2780 (A and B), A2780/ADR (C and D) and A2780/CP (E and F). Data collected from triplicates at 15 min intervals are expressed as cell index (A, C and E) or cell index normalized to 6-h post-plating (B, D and F). Slope of cell index for all three cell lines is illustrated in (G). These data show that SFN reduced cell migration without affecting cell proliferation.

Finally, we tested whether SFN affects behavior of A2780 cells and of the chemoresistant variants. Since tumor progression to metastasis is associated with hypoxia and induction of migratory phenotype (25), we examined the ability of SFN to modulate migration of A2780 variants. To this end we used an impedance-based approach, which allows for the real-time evaluation of cell migration. We found that SFN reduces migration of hypoxic chemosensitive and chemoresistant A2780 cell lines (Fig. 6A, C, E and G). To exclude the contribution of cell proliferation to the observed effect, we simultaneously monitored this parameter by real-time measurement of the cells plated in parallel, pre-treated in hypoxia and grown on the bottom of impedance plates. This control experiment showed no differences in the proliferation cell index of the analyzed cell lines (Fig. 6B, D and F). Thus, we can conclude that SFN can diminish migration of the ovarian carcinoma cells that were exposed to hypoxia.

## Discussion

As an attractive opportunity for cancer prevention and treatment, sulforaphane has been extensively studied in the context of its anticancer effects and targets. Although the list of the SFN studies is large, most of them suffer from one of two drawbacks. Firstly, SFN was often evaluated in high concentrations that cannot be reached *in vivo* (10,12,26). Secondly, the vast majority of studies was performed in normoxia, although almost all solid tumors contain hypoxic areas, which are known to affect cell behavior and reduce response to therapies (8–10,22,26). Actually, there are only a handful of studies describing the effects of SFN in hypoxic tumor cells (12,13,27).

Herein, we used the hypoxic A2780 ovarian cancer cells as a model. Ovarian tumors are characterized by the presence of hypoxia, aggressive behavior and resistance to therapy. Hypoxia affects the phenotype of ovarian cancer cells by inducing stem-like properties (17), migration and invasion (28) and reduced sensitivity to drugs (29,30). Moreover, hypoxia correlates with poor prognosis and weak response to therapy in ovarian cancer patients (4,31,32). In some of these studies, treatment-refractory tumors display expression of the hypoxia-induced CA IX, which is associated with advanced cancer stages and poor clinical outcome (18,33–35).

We focused on the effects of SFN at 5 μM, a concentration matching with the bioavailable levels in cells exposed to moderate hypoxia. This dose was eight-times lower than that used by Yao *et al* (12), who did not show the viability data. However, based on the other reports, SFN increases cell death in hypoxic tumor cells, especially at higher doses (13,36). On the other hand, Chen *et al* (22) used SFN at 5 μM concentration in normoxia and obtained only minor effect on cell survival.

In our study, moderate hypoxia slightly reduced the survival of A2780 cells and 5 μM SFN showed only a very modest effect. However, at this concentration SFN inhibited the HIF-related pathway and AP-1-mediated transcriptional activation. Since both pathways contribute to the hypoxic signaling, SFN clearly affects molecular responses to hypoxia. Simultaneously, SFN activated the pathways that negatively control tumor growth and usually interfer with or are suppressed by hypoxia. For example, hypoxia reduces the level and transcriptional activity of the wt p53 (37), whereas SFN activates the wt p53 reporter as shown here and elsewhere (38). Similarly, NRF2 is usually activated as an antioxidant response and is modulated by hypoxia (39). However, SFN induces this antioxidant pathway as was shown also in other studies using different cellular models (40).

Furthermore, in A2780 cells SFN also induced the transcriptional response via the xenobiotic-responsive element XRE that activates the expression of drug-metabolizing enzymes. Such XRE-mediated response has been associated with SFN in several cell types (10). This pathway is usually downregulated by hypoxia (41,42), so it is plausible that it would be upregulated with the decrease of the hypoxic signaling. SFN increased transcriptional activation by IRF-1 (IFN-regulatory factor 1) acting as a tumor suppressor binding to upstream regulatory regions of IFN-1 and IFN-1-induced MHC class I genes (43). Thereby SFN can support the immune response against tumor cells and contribute to reduced tumor growth. All of these pathways were individually associated with SFN in earlier studies, but here we detected they simultaneous modulation, in agreement with the known pleiotropic anticancer activity of SFN.

The most prominent change induced by SFN was the down-regulation of the HIF-1-mediated transcriptional response through a diminished expression of the HIF-1α protein, the HIF-1 subunit sensitive to oxygen. Since we did not observe any changes in the HIF-1α transcription and degradation, we propose that SFN affects its translation. This is in agreement with data of Yao *et al* (12) in hypoxic carcinoma cells derived from tongue and prostate. The role of SFN in regulation of the translational machinery in hypoxia has not been thoroughly investigated so far, however, its inhibitory effect on mTOR pathway was shown in PC3 prostate cancer cells cultivated under standard conditions (44). Thus, we assume that the ability of SFN to interfere with the protein synthesis might represent a general phenomenon.

HIF-1 trans-activates a multitude of genes mediating adaptive responses to hypoxia. The *CA9* gene is one of the most strongly activated HIF-1 downstream targets, because the HIF-1-responsive element HRE is localized next to its transcription start site (45). Moreover, AP-1-responsive element is localized just upstream of HRE and contributes to *CA9* transcription (46). On the other hand, p53 was shown to reduce the HIF-1-mediated induction of CA IX in connection with the DNA damage response in hypoxic cells (47). Similarly, XRE element was shown to downregulate the hypoxic induction of CA IX (42). Thus it is not surprising that SFN reduced the *CA9* promoter activation and transcription and the CA IX protein level. Of note, SFN also decreased proportion of the CA IX-positive cells in the population of A2780 cells suggesting that it eliminated the subpopulation that was most responsive to hypoxia. Given that hypoxia promotes the aggressive tumor cell phenotype, SFN appears to predominantly target the adaptable hypoxic cells.

CA IX is a cell surface enzyme catalyzing the conversion of carbon dioxide to bicarbonate ions and protons (48) and regulating pH in hypoxic tumor cells (21,49). This represents an adaptation to the hypoxia-triggered oncogenic metabolism largely relying on glycolysis and generating acidic metabolic products that have to be eliminated from tumor cells to preserve their intracellular pH and protect their survival (50). CA IX helps to accomplish this acid extrusion by speeding up the bicarbonate production and import through the bicarbonate transporters and consequently export of CO_2_ and protons to the extracellular milieu. Thereby it contributes to pericellular acidosis, which supports cell migration and invasion (21). Thus, it is not surprising that SFN, via suppressive effect on the CA IX expression and pH regulation, decreases the migration of A2780 ovarian carcinoma cells.

An important finding of this study is that SFN elicited similar effects (resulting in the downregulation of the HIF-1α and CA IX protein levels and in the decreased migration) also in chemoresistant cell lines under hypoxic conditions, namely in cisplatin-resistant A2780/CP cells and adriamycin-resistant A2780/ADR cells. Both hypoxia (and HIF-1α pathway) and CA IX protein have been associated with chemoresistance in various tumor types (51,52). The effects of hypoxia on resistance of tumors to chemotherapy can be attributed to: a) reduced diffusion of drugs to hypoxic areas; b) decreased proliferation of hypoxic tumor cells due to HIF-1-induced energy-saving pathways; c) selection of inherently resistant cells with mutations in the DNA damage response; d) HIF-1-mediated activation of the DNA repair apparatus, e) HIF-1-triggered induction of genes conferring drug resistance, and f) HIF-1-triggered death of cells unable to adapt to hypoxia. Whereas diffusion distance and selection processes are not relevant for the experiments in the monolayer culture, our experimental approach still involves the anti-proliferative and death-inducing effects, and adaptations modulating tumor cell phenotype.

Noteworthy, the hypoxia-induced promoter activation of the *CA9* gene was higher in the chemoresistant cell variants compared to the parental A2780 cells (see the Figs. 3A vs. 4B) suggesting that chemoresistant cells are more responsive and/or adaptable to changes in oxygen levels. On the other hand, SFN (at its bioavailable concentration) was able to diminish the expression of both HIF-1α and CA IX, although the decrease did not reach the normoxic values. Thus, we assume that SFN can partially overcome the chemoresistance of ovarian cancer cells. This offers a path that can be potentially exploited in sensitizing resistant cancer cells to therapy, and opens a window for combined drug treatments of SFN either with chemotherapeutic drugs or with natural compounds. There are several examples showing improved anticancer effects of SFN combined with other compounds (20,22,53,54). Of note, simultaneous treatment with SFN and acetazolamide, a pan-carbonic anhydrase inhibitor that also inhibits CA IX, was very effective in the bronchial carcinoid cell lines both *in vitro* and *in vivo* (55). Our study may not only provide the explanation for these observations but also show new directions for the rational application of SFN in anticancer strategies against hypoxic tumors.

## Abbreviations

ADR: adriamycin
AP-1: activator protein 1
ARE: antioxidant-response element
CA IX: carbonic anhydrase protein
*CA9*: human carbonic anhydrase gene
CP: cisplatin
GLUT: glucose transporter
HIF: hypoxia-inducible factor
HRE: hypoxia-response element
IRF-1: interferon-regulatory factor 1
JNK: Jun N-terminal kinase
MAPK: mitogen activated protein kinase
pHi: intracellular pH
pHe: extracellular pH
SFN: sulforaphane
SP-1: specificity protein 1
VEGF: vascular endothelial growth factor
VHL: von Hippel Lindau
XRE: xenobiotic-response element
