# [Corrigendum] Sulforaphane reduces molecular response to hypoxia in ovarian tumor cells independently of their resistance to chemotherapy

**DOI:** 10.3892/ijo.2026.5931

**Published:** 2026-08-20

**Authors:** Michal Pastorek, Veronika Simko, Martina Takacova, Monika Barathova, Maria Bartosova, Luba Hunakova, Olga Sedlakova, Sona Hudecova, Olga Krizanova, Franck Dequiedt, Silvia Pastorekova, Jan Sedlak

Int J Oncol 47: 51-60, 2015; DOI: 10.3892/ijo.2015.2987

Following the publication of the above article, an interested reader drew to the Editor's attention that the β-actin control protein band featured in the right-hand lane of Fig. 1A on p. 54 appeared to be strikingly similar to the β-actin control protein band featured in the right-hand lane of Fig. 2B on the same page, albeit after vertical flipping and with possible horizontal and vertical resizing. Owing to the lack of availability of the raw western blot data due to the time that has elapsed since this paper was published, the authors have repeated the experiments and provided new western blots for these figures, which fully confirm the veracity of the findings presented in the originally published study. The revised versions of Figs. 1 and 2 are shown on the next page.

In response to an additional query, the authors also wished to point out that the raw data from the PCR analysis (which were presented to the Editorial Office for inspection) confirmed that the data were correctly assembled from the original electropherograms, and that the part of Fig. 4C associated with the A2780/ADR cells was composed of two parts that came from the same experiment and the same run. They were put together as such, simply because they were loaded in two separate parts of the gel. The authors thank the Editor of *International Journal of Oncology* for granting them the opportunity to publish this corrigendum. All the authors agree with the publication of this corrigendum; furthermore, they apologize to the readership of the journal for any inconvenience caused.

## Figures and Tables

**Figure 1:**
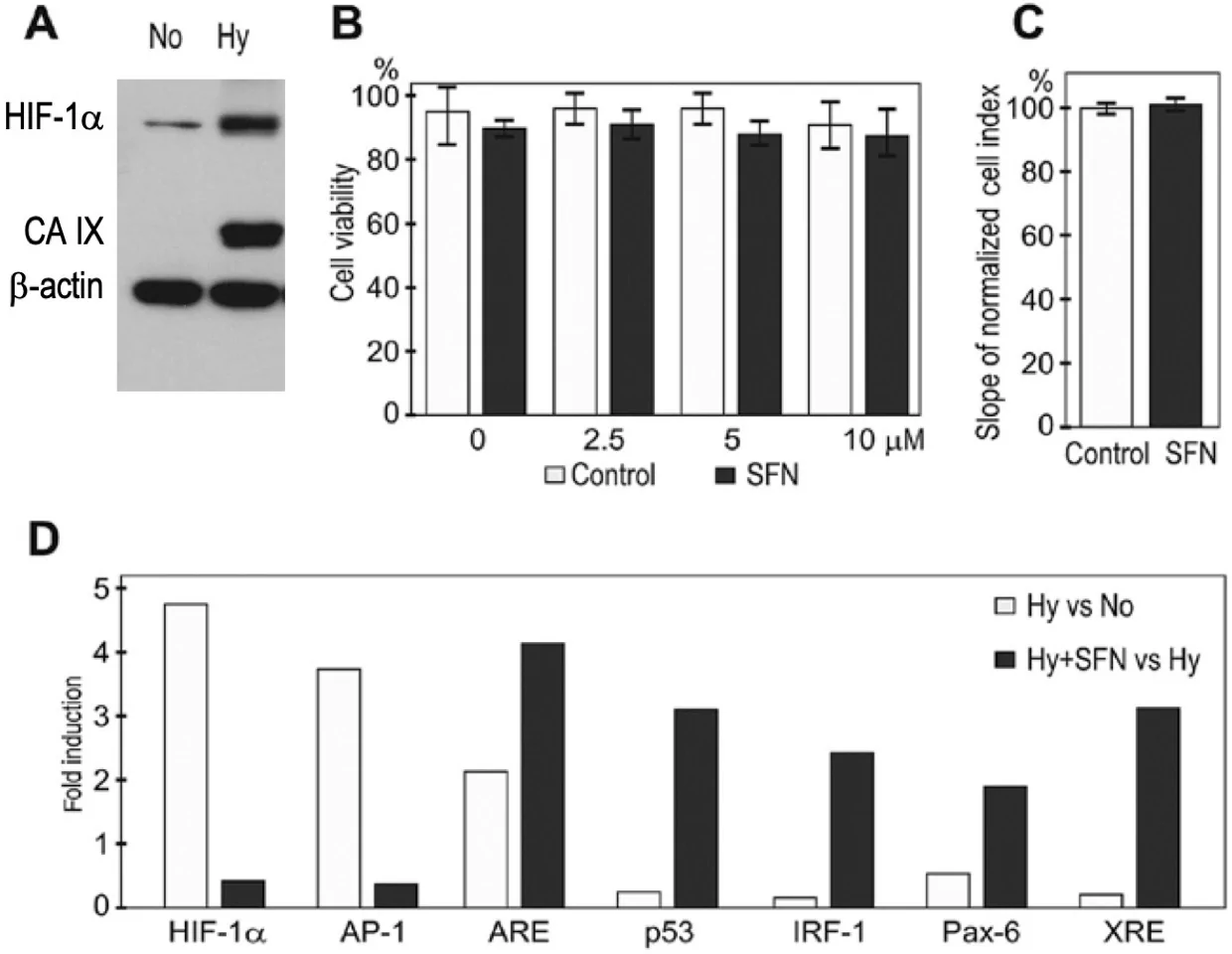
Molecular and cellular response of A2780 cells to hypoxia and SFN. (A) Western blot analysis of HIF-1α and CA IX expression in A2780 cells. The blot shows that these proteins are absent in normoxia (No) but induced in response to hypoxia (Hy). Actin serves as a loading control. (B) Flow cytometric analysis of the viability of hypoxic vs. normoxic A2780 cells (control) and the parallel normoxic vs. hypoxic samples treated with increasing concentrations of SFN as described in Materials and methods. The graph depicting percentage of live (propidium iodide-negative) cells in population indicates only insignificant decrease in cell viability in all treated samples. (C) Real-time analysis of proliferation of the hypoxic A2780 cells treated with 5 μM SFN for 48 h compared with the non-treated controls. The graph shows the slope of cell index normalized to 6 h time point after plating at which time all cells were attached to the bottom of the wells of the impedance plate and hypoxia was settled at 2%. Data indicate that SFN treatment did not affect the proliferation of hypoxic A2780 cells. (D) Cell-based dual luciferase reporter assay of hypoxic vs. normoxic A2780 cells and hypoxic A2780 cells vs. cells treated with 5 μM SFN revealed hypoxia- and SFN-induced alterations of several signal transduction pathways leading to changes in transactivation activities of the transcription factors indicated in the graph. In principle, SFN downregulated pro-oncogenic pathways (represented by HIF-1α and AP-1) and upregulated anti-oncogenic pathways (such as ARE/NRF2, p53, IRF-1, Pax-6 and XRE).

**Figure 2:**
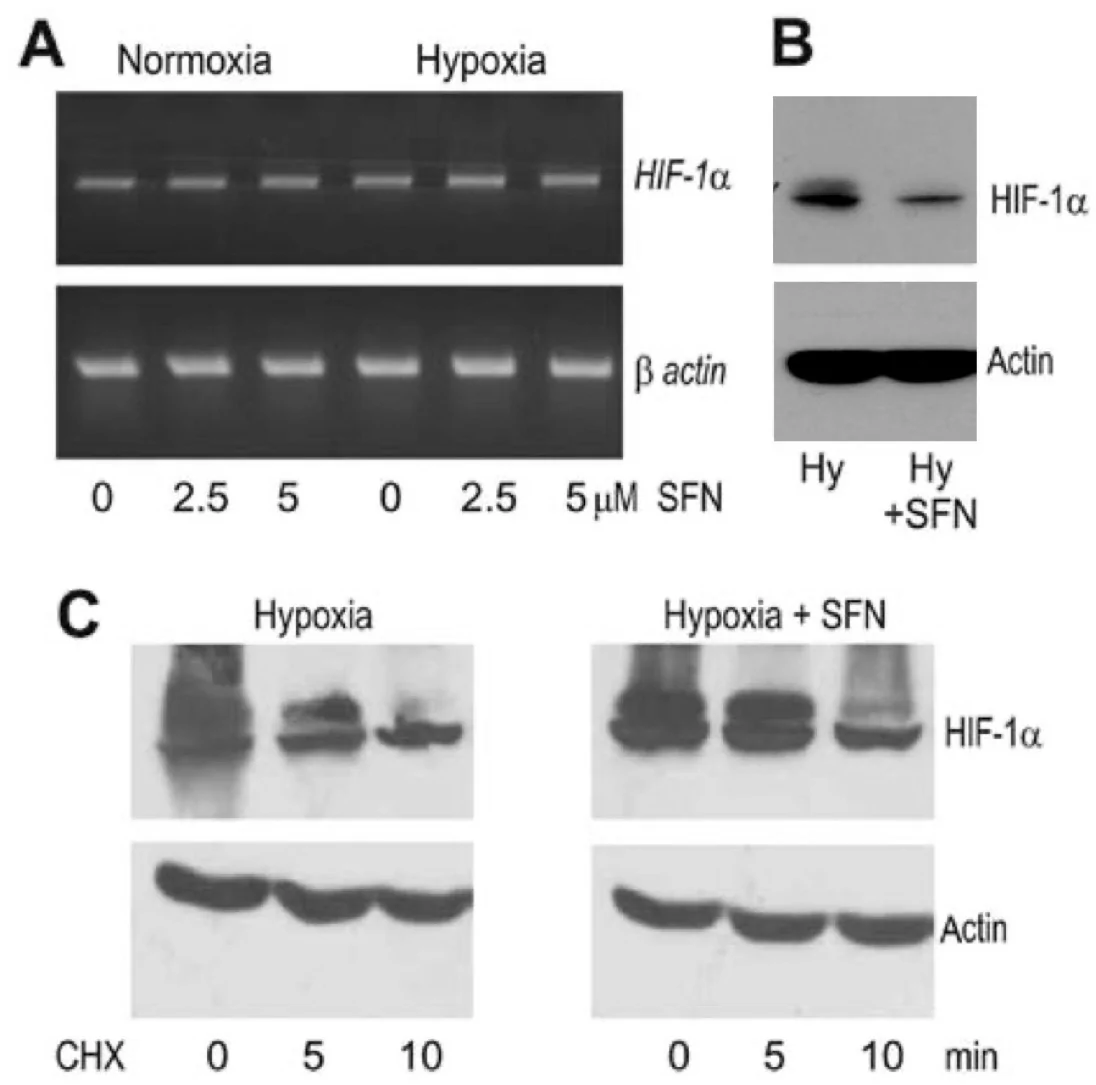
Effect of SFN on HIF-1α transcription, protein level and stability in A2780 cells. (A) Reverse-transcription PCR analysis of the HIF-1α transcription in normoxic and hypoxic A2780 cells in the absence and the presence of SFN. β-actin was used as standard. SFN treatment did not change the levels of the HIF-1α transcript. (B) Western blot analysis of HIF-1α protein levels in non-treated hypoxic A2780 cells (Hy) and in hypoxic A2780 cells treated with 5 μM SFN (Hy+SFN). (C) Western blot analysis of HIF-1α protein stability in non-treated (Hypoxia) and in 5 μM SFN-treated hypoxic A2780 cells (Hypoxia+SFN) in the presence of 20 μg/ml cycloheximide (CHX). SFN treatment did not affect the HIF-1α level suggesting that it did not decrease the HIF-1α protein stability.versus control cells.

